# Effects of Docosahexanoic Acid on Gut Microbiota and Fecal Metabolites in HIV-Infected Patients With Neurocognitive Impairment: A 6-Month Randomized, Double-Blind, Placebo-Controlled Trial

**DOI:** 10.3389/fnut.2021.756720

**Published:** 2022-01-21

**Authors:** Ruihua Dong, Haijiang Lin, Yingying Ding, Xiaoxiao Chen, Ruizi Shi, Shiying Yuan, Jing Li, Bowen Zhu, Xiaohui Xu, Weiwei Shen, Keran Wang, Ding Ding, Na He

**Affiliations:** ^1^Department of Nutrition and Food Hygiene, School of Public Health, Fudan University, Shanghai, China; ^2^Department of Epidemiology, School of Public Health, and the Key Laboratory of Public Health Safety of Ministry of Education, Fudan University, Shanghai, China; ^3^Taizhou City Center for Disease Control and Prevention, Taizhou City, China; ^4^Institute of Neurology, Huashan Hospital, Fudan University, Shanghai, China; ^5^Shanghai Institute of Infectious Diseases and Biosafety, Fudan University, Shanghai, China

**Keywords:** docosahexanoic acid (DHA), HIV, neurocognitive impairment (NCI), gut microbiota, fecal metabolites

## Abstract

Neurocognitive impairment (NCI) and gut microbiota dysbiosis are prevalent in patients with HIV infection. Docosahexanoic acid (DHA) supplementation may alleviate multiple neurocognitive diseases symptoms and plays important role in regulating gut microbiota. However, it is not known whether DHA algae oil supplements can alleviate neurocognitive impairment (NCI) and regulate gut microbiota and fecal metabolites. A randomized, double-blind, placebo-controlled trial was performed on 68 HIV-infected patients with NCI. Participants were randomized to receive a 3.15 g daily DHA algae oil supplement or placebo for 6 months. We collected blood and fecal samples from these patients before and after the trial. Mini mental state examination (MMSE) and neuropsychological tests (NP tests) were administered to assess the cognitive status of participants. The influence of DHA algae oil on the gut microbiota, fecal metabolomics, plasma proinflammatory, and oxidative stress factors was also investigated. There were no significant changes in NCI according to global diagnosis score (GDS) and MMSE score within the two groups, while patients receiving DHA had improvement in several blood lipids, pro-inflammatory and oxidative stress factors. The DHA supplement increased α-diversity indexes, increased abundances of *Blautia, Bifidobacterium, Dorea, Lactobacillus, Faecalibacterium, Fusobacterium*, and *Agathobacter*, and decreased abundances of *Bacteroides* and *Prevotella_9*. Furthermore, DHA supplement was correlated with improved fecal lipid metabolites as indicated by ceramides, bile acids, glycerophospholipids. In addition, the DHA supplement was associated with altered cholesterol metabolism and purine metabolism pathways. A daily supplement of DHA algae oil for 6 months has been shown to promote favorable transformations in gut microbiota, profiles of fecal metabolomic, and factors responsible for proinflammatory and oxidative stress, which might be beneficial for the prognosis of HIV-infected patients with NCI in the long-term.

**Clinical Trial Registration:**

https://clinicaltrials.gov/ct2/show/NCT04242004, identifier: NCT04242004.

## Introduction

Patients infected with HIV are prone to neurocognitive impairment (NCI) (1). The NCI symptoms in HIV-infected individuals may be derived from the HIV infection, side effects of specific antiretroviral drugs (ARVs), or the aging of the HIV population (2). Gut microbiota dysbiosis is common in HIV-infected individuals, including decreased diversity and alterations in gut microbiome composition compared with healthy individuals (3). Growing evidence reveals that gut microbiota has a great influence on the pathogenesis of neurocognitive diseases (4, 5). Thus, gut microbiota may be considered as a possible target to treat NCI.

N-3 polyunsaturated fatty acids (n-3 PUFAs) can inhibit inflammatory responses (6). Accumulating experimental studies have been published about the positive effect of n-3 PUFAs on the gut microbiota (7). However, the impact of n-3 PUFAs on the human microbiome has been less well-defined. Given the influence of n-3 PUFAs on microbiota and its anti-inflammatory effects, supplementation with n-3 PUFAs can be a potential intervention or therapy for many neurocognitive diseases (8). In the HIV setting, many clinical trials have focused on the effect of n-3 PUFAs on the CD4 cell count and the level of triglycerides (TGs) (9–11). For example, n-3 PUFAs supplementation can reduce TG levels and increase CD4 cell count (12–14). However, there is a lack of clinical trials that evaluate the effect of n-3 PUFAs on NCI among HIV-infected individuals, as well as the gut microbiota. Thus, we hypothesized that supplementation with n-3 PUFAs might alleviate the NCI symptoms associated with HIV infection, acting through gut microbiota regulation.

Human data have shown that different n-3 PUFAs have different effects on cardiometabolic health markers (15). However, most clinical trials were studying n-3 PUFAs as a mixture, which has presented controversial results about whether n-3 PUFAs can improve cognitive function. Docosahexanoic acid (DHA) is a principal n-3 PUFAs extracted from marine oils, required as a structural component of the brain, and is essential for numerous brain functions (16). Increased DHA consumption can reduce the risk of depression, schizophrenia, bipolar disorder, and other brain disorders in different populations (17–19). Moreover, previous studies showed that the level of DHA was decreased in the blood of HIV-infected patients (20). Nonetheless, it remains to be investigated whether DHA supplementation can change the cognitive status of HIV-infected patients. To fill this gap, we carried out a randomized, double-blind, placebo-controlled clinical trial to evaluate the effect of DHA algal oil on NCI among patients infected with HIV who received combination antiretroviral therapy (cART), as well as the change of gut microbiota, profiles of fecal metabolomics, and plasma biomarkers of proinflammatory responses and oxidative stress.

## Methods

### Participants and Study Design

The trial was conducted in a double-blind, randomized, placebo-controlled manner. HIV-infected patients were recruited through the Comparative HIV and Aging Research in Taizhou (CHART). The detail of the prospective cohort has been described previously (21). In brief, we screened 126 HIV-infected adults. The inclusion criteria for this trial require male and female patients diagnosed with HIV-1 infection and were under stable cART (6 months prior and within the study period) and diagnosed with NCI. The presence of NCI was diagnosed according to the global diagnosis score (GDS) from a set of neuropsychological tests (NP tests) (22). Exclusion criteria included patients taking drugs with psychic effects, patients with a value of BMI >30 kg/m^2^, women during lactation or pregnancy, patients with diabetes mellitus history, cardiovascular and cerebrovascular diseases, or any other diseases in the liver, kidney, and hematopoietic system.

Finally, 88 eligible participants were randomized in 1:1 to receive daily algal oil DHA supplement (Supplementary Table 1) or a matching placebo of soy oil capsules. Each capsule provided 450 mg DHA. Doses were 3.15 g/day. The treatment duration was 24 weeks. Randomization was done according to a computer-generated random schedule. Both investigators and participants were blind to the intervention condition while assessing outcome measures before the completion of data analysis. All participants received instructions on maintaining their current diets and physical activities throughout the intervention period. All participants were contacted at weeks 4, 8, 12, 16, and 20 to evaluate any problems that might occur due to the drugs studied. After 24 weeks of intervention, 68 participants (77.3%) completed the whole trial.

Study visits were undertaken on day 1 (baseline) and week 24 (final follow-up). At each visit, blood and fecal samples were collected. All participants were administered with a questionnaire interview to collect dietary intake, basic demographics, and lifestyle. We used a valid food frequency questionnaire (FFQ) for dietary assessment (23). Current smoking and alcohol use were defined as having smoked and drink in the last 30 days (22). The main outcome of this study was mental status which was assessed by two questionnaires. Face-to-face neurocognitive testing was conducted on all participants by trained health staff according to both the MMSE in mandarin (1) and the NP tests. Additionally, they received comprehensive physical and biochemical examinations and B-mode ultrasound examinations. Carotid intima-media thickness (CIMT) was imaged using a high-resolution B-mode ultrasound machine with a 10 MHz multi-frequency linear transducer (LOGIQ P5 pro, General Electric Medical Company, WI, USA). A standardized protocol was used for images and procedures. This study was approved by the Institutional Review Board (IRB) of the School of Public Health at Fudan University (no.IRB#2019-06-0759), Shanghai, China. All individuals gave informed consent at enrollment. The registration of this study can be found in ClinicalTrials.gov (no. NCT04242004).

### Assessment of Compliance

Participants were asked to record acceptability and adverse events. The research Staff inquired the participants about whether they consumed the assigned capsules and how many capsules they consumed each day during routine follow-up communications (once a month). Compliance with the supplementation was monitored by counting the capsules returned by the participants. The adherence to the intervention was defined as a percentage; (number of capsules supplied a minus number of capsules not taken)/number of capsules supplied × 100. Participants consumed a mean of 94.6% of the packets (mean of 94.6% in the DHA group and mean of 94.7% in the placebo group).

### Neuropsychological Tests

Multiple cognitive-motor ability domains that were contently found in HIV-associated brain disease in patients from the United States were tapped in the test battery. The battery was carefully reviewed and approved to be culturally appropriate for the study populations in China by mental health professionals (22). Ten NP tests were used to evaluate seven domains: semantic verbal fluency (animals); Hopkins Verbal Learning Test; Brief Visuospatial Memory Test; Stroop Color and Word Test; Trail Making Test (parts A and B); Wisconsin Card Sorting Test 64 Card Version (WCST-64); Grooved Pegboard Test; Paced Auditory Serial Addition Test (PASAT); the Digit Symbol, Symbol Search, and Letter-Number Sequencing tests from the Wechsler Adult Intelligence Scale (WAIS-III).

Scaled scores were converted from raw test scores from these tests. For each test, standardized T scores were generated with the mean of 50 (SD, 10) and adjusted for age, sex, and education. Based on T scores, deficit scores (DSs) ranging from 0 (no deficit) to 5 (severe deficit) were created: T score ≥ 40 = DS score of 0; T score 35–39 = DS score of 1; T score 30–34 = DS score of 2; T score 25–29 = DS score of 3; T score 20–24 = DS of 4; T score <20 = DS score of 5. To generate global T and GDS, domain T and DS took the average scores of individual tests within each domain and across all tests, respectively. The global cognitive score was defined by the GDS dichotomized as impaired (GDS ≥ 0.5) or unimpaired (GDS < 0.5).

### Collection of Blood Specimen and Analysis of Proinflammatory and Oxidative Stress Factors

Serum samples were taken from the supernatant of blood after centrifugation and were immediately stored at −80°C for further analysis. As described previously, the clinical biomarkers, including glucose (Glu) and lipid profiles [total cholesterol (TC), TG, low-density lipoprotein (LDL), and high-density lipoprotein (HDL)], were measured (24). Concentrations of plasma DHA, proinflammatory factors, including tumor necrosis factor-α (TNF-α), interleukin 6 (IL-6), soluble CD14 (sCD14), and high-sensitive C-reactive protein (hs-CRP) and oxidative stress factor, including 8-F2c-isoprostane and malondialdehyde (MDA) were measured using ELISAs kits (Shanghai Enzyme-linked Biotechnology Co., Ltd., Shanghai, China) according to the instructions of the manufacturer (Supplementary Table 2). The precision of the assay was confirmed for a low coefficient of variations, <10% for intra-assay and inter-assay.

### Analysis of Fecal Metabolomics With 16S rRNA Sequencing

The fecal samples were collected in a feces container and stored at −80°C immediately upon collection. Total genomic DNA was isolated using DNA Extraction Kit (Qiagen, Düsseldorf, Germany) as per instructions from the manufacturer. Details of the quantitative measurement of fecal metabolomics using 16S rRNA sequencing as well as relevant data analysis can be found in Supplementary Materials.

### Statistical Analyses

The sample size was calculated on the basis of the MMSE. With a power of 80% and a type I error of 5%, a sample size of 36 per group would be required to detect an effect size of the change in 0.5 SD between groups (1). Allowing for a 10% loss during over 6 months of intervention, a sample size of 40 per group was considered adequate. In the end, 34 participants per group completed the whole trial.

Comparisons of clinical parameters, demographic factors, and dietary intake between the two groups at baseline were included in the descriptive statistics analysis. According to the distribution of data, median (interquartile range [IQR]) or mean (SD) were used to describe continuous variables. Categorical variables were summarized as numbers and percentages (%). Differences for 24 weeks changes from baseline of clinical parameters, proinflammatory and oxidative stress markers were analyzed using Student's *t*-test or Mann–Whitney *U*-test according to the distribution of data.

To explore the role of gut microbiota and the underlying metabolic mechanisms, we compare the gut microbiota and profiles of fecal metabolomics before and after DHA intervention. The α-diversity indices, including Chao, Shannon, and Simpson, were calculated. Beta diversity was measured by unweighted UniFrac distance. Differences in characteristics and diversity indices between groups were analyzed using the Mann–Whitney *U*-test (continuous variables and skewed distribution), the Student's *t*-test (continuous variables and normal distribution), or the chi-square test (categorical variables). Wilcoxon rank-sum test was employed to identify the differential bacterial genera abundance between the two groups. Correlations between changes in clinical parameters, proinflammatory and oxidative stress markers, and alterations in genus relative abundance, were evaluated with Spearman's rank.

To investigate the overall microbial metabolites distribution between the two groups, orthogonal projections to latent structure-discriminant analysis (OPLS-DA) were performed. The fit and predictability of the models obtained were determined by the *R*^2^ Y and *Q*^2^ values, respectively. In the OPLS-DA model, we generated the variable importance plot (VIP) to select potential biomarkers. In biochemical pathways of predicted Kyoto Encyclopedia of Genes and Genomes (KEGG), a Wilcoxon matched-pairs test was adopted. To analyze the association between fecal metabolites and genus relative abundance, as well as clinical parameters, proinflammatory cytokines, and oxidative stress markers were analyzed using Spearman's rank test. A *p* < 0.05 was defined as statistically significant. To control the false discovery rate (FDR), the values of *p* were adjusted. All statistical analyses were conducted using R version 3.4.

## Results

### Baseline Characteristics and Subject Disposition

**Figure 1:**
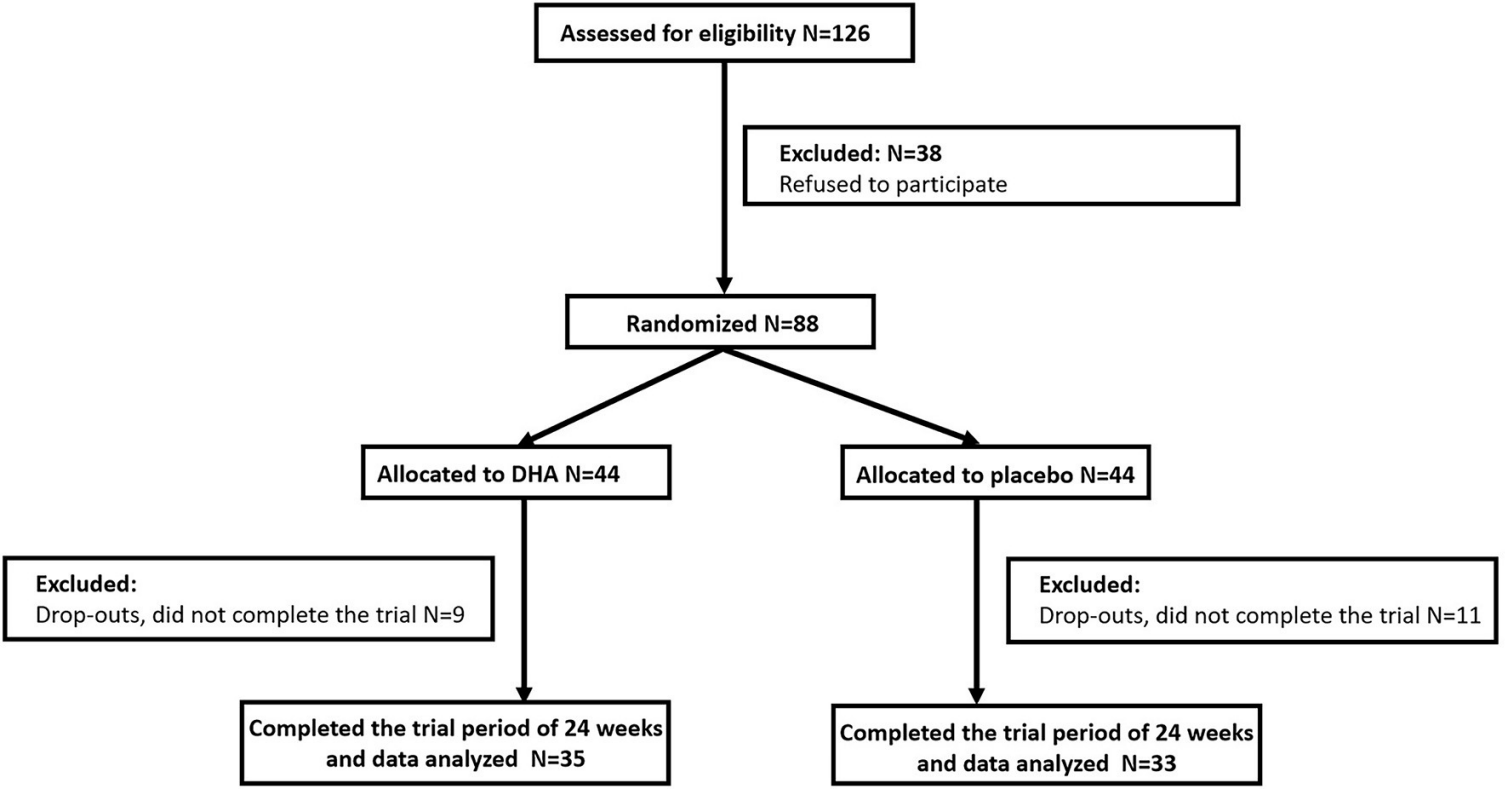
Subject disposition.

We selected 88 subjects and grouped them in a randomized manner and 68 (77.3%) completed the evaluation for this 24-week study (Figure 1). Patient characteristics at baseline are summarized in Table 1. We found no significant differences in sex, age, and BMI between the two groups of participants. DHA group had a lower LDL level compared with the placebo group, while other clinical parameters and the intakes of dietary components had no difference between the two groups from the beginning of this study.

**Table 1:** Baseline characteristics of the participants (*n* = 68).

**Characteristics**	**DHA group** **(*n* = 35)**	**Placebo group** **(*n* = 33)**	***P-*values**
**Demographics**			
Sex, *n* (%)			
Male	30 (85.7)	26 (78.8)	0.454
Female	5 (14.5)	7 (21.2)	
Age, years (mean ± SD)	54.6 ± 9.7	55.3 ± 9.3	0.708
BMI, kg/m^2^ (mean ± SD)	23.2 ± 3.0	22.9 ± 2.6	0.679
**Education**, ***n*** **(%)**			
≤ Primary school	24 (58.6)	22 (66.6)	***0.036*** ^*^
middle school	10 (28.6)	6 (18.2)	
High school	1 (2.9)	5 (15.2)	
**Exercise**, ***n*** **(%)**			
Yes	9 (25.7%)	15 (45.5%)	0.089
No	26 (74.3%)	18 (54.5%)	
**Current smoker**, ***n*** **(%)**			
Yes	14 (40.0%)	18 (54.5%)	0.230
No	21 (60.0%)	15 (45.5%)	
**Current alcohol use**,***n*** **(%)**			
Yes	18 (51.4 %)	14 (42.4%)	0.457
No	17 (48.6 %)	19 (57.6%)	
Current CD4 count, cells/μL (median, IQR)	381.0 (226.0, 518.0)	387.0 (293.0, 593.0)	0.287
**Clinical parameters**			
HDL, mmol/L (mean ± SD)	1.2 ± 0.5	1.3 ± 0.3	0.341
LDL, mmol/L (mean ± SD) TC, mmol/L (mean ± SD)	1.8 ± 0.8 4.3 ± 1.1	2.5 ± 0.7 4.7 ± 0.8	0.001^*^ 0.076
TG, mmol/L (median, IQR)	2.1 (1.2, 2.9)	1.8 (1.1, 2.8)	0.207
WBC, 10^9^ cells/L (median, IQR)	5.2 (4.2, 6.5)	4.7 (4.2, 5.5)	0.413
Left CIMT, mm (median, IQR)	1.1 (0.7, 1.4)	0.9 (0.8, 1.0)	0.070
Right CIMT, mm (median, IQR)	1.2 (0.8, 1.2)	0.9 (0.8, 1.0)	0.125
**Oxidative stress markers**			
8-sio-PGF2α, pg/ml	3228.0 (3017.3, 4593.3)	3620.0 (3064.0,4065.3)	0.394
MDA, nmol/ml	17.2 (12.8, 20.4)	16.3 (12.7, 20.4)	0.451
**Proinflammatory markers**			
TNF-α, ng/L	430.6 (364.7, 532.7)	411.1 (352.9, 513.7)	0.366
hs-CRP, μg/L	2071.7 (1775.5, 2830.5)	1532.7 (1291.9, 1945.5)	0.564
sCD14, μg/L	51.9 (41.4, 60.9)	47.5 (42.5, 62.0)	0.339
IL-6, ng/L	19.1 (16.5, 24.8)	16.4 (14.0, 22.1)	0.421
MMSE	27.0 (22.0, 29.0)	27.0 (25.0, 28.0)	0.579
GDS	0.3 (0.03, 0.6)	0.4 (0.01, 0.6)	0.462
^**a**^ **Dietary nutrient**			
Energy, kcal/d	2783.1 (1745.5, 3225.3)	1879.7(1146.8, 3342.4)	0.059
Carbohydrate, g/d	369.0 (282.7, 578.9)	283.8 (180.7, 522.9)	0.062
Protein, g/d	108.9 (59.5, 131.6)	76.6 (45.7, 139.9)	0.097
Fat, g/d	56.8 (32.6, 96.2)	35.5 (24.0, 60.5)	0.172
Dietary fiber, g/d	18.9 (10.8, 27.1)	13.2 (10.4, 24.6)	0.448
Cholesterol, g/d	320.1 (173.7, 556.2)	254.5 (165.4, 484.1)	0.646
DHA, ng/L	56.9 (46.6, 66.7)	50.9 (41.9, 65.4)	0.550

cART, combination antiretroviral therapy; BMI, body mass index; HDL, high-density lipoprotein; LDL, low-density lipoprotein; TC, total cholesterol; TG, triglyceride; CIMT, carotid intima-media thickness; WBC, white blood cells; TNF-α, tumor necrosis factor-α; IL-6, interleukin 6; sCD14, soluble CD14; hs-CRP, high-sensitive C-reactive protein; 8-sio-PGF2α, 8-F2α-isoprostane; MDA, malondialdehyde; MMSE, the Chinese version of Mini-mental State Examination score; GDS, global deficit score; IQR, interquartile range; SD, standard deviation; DHA, docosahexanoic acid; Bold italic ^*^p < 0.05; ^a^distribution of dietary nutrients according to the food frequency questionnaire survey.

### Effects of DHA Supplement on the Markers of NCI, Blood Lipid, Inflammatory, and Oxidative Stress

Anthropometric data, GDS, MMSE score, and clinical parameters are summarized in Table 2. There were no significant changes in NCI according to GDS and MMSE score within the two groups after the 6-month intervention. After the 24 weeks intervention, levels of TG and WBC in the DHA group were significantly reduced than those observed in the placebo group. Moreover, the level of LDL was significantly increased compared with that observed in the soy group. There were no significant changes in other clinical parameters between the two groups. Additionally, compared with the placebo group, levels of TNF-α and hs-CRP were significantly reduced in the DHA group (Table 2). There were no significant changes in dietary intake (Supplementary Table 3).

**Table 2:** Effects of DHA supplement on concerned parameters (*n* = 68).

**Characteristics**	**DHA group (*n* = 35)**	**Placebo group (*n* = 33)**	***P-*values**
DHA, ng/L	23.6 (11.1, 31.8)	−5.3 (−17.9, 10.8)	***<0.001*** ^***^
**NCI**			
GDS	0.2 (0.1, 0.5)	0.1 (−0.1, 0.6)	0.646
MMSE	0.0 (−2.0, 1.0)	−1.0 (−2.0, 0.5)	0.658
**Clinical parameters**			
Current CD4 count, cells/μL	26.5 (−37.2, 102.3)	14.0 (−40.0, 92.5)	0.773
WBC, 10^9^ cells/L	−5.0 (−1.0, 0.4)	0.3 (−0.4, 1.4)	***0.022*** ^*^
TG, mmol/L	−0.41 (−1.73, −0.10)	−0.09 (−0.58, 0.39)	***0.004*** ^**^
TC, mmol/L	0.04 (−0.42, 0.56)	−0.09 (−0.44, 0.43)	0.669
HDL, mmol/L	−0.03 (−0.23, 0.04)	−0.12 (−0.24, 0.06)	0.101
LDL, mmol/L	0.18 (−0.25, 0.58)	−0.38 (−0.77, 0.06)	***0.001*** ^**^
**Oxidative stress markers**			
8-sio-PGF2α, pg/ml	−1840.1 (−2948.3, −1390.8)	−2063.3 (−2834.2, −1618.7)	0.763
MDA, nmol/ml	−9.4 (−12.6, −3.7)	−8.9 (−12.3, −5.9)	0.439
**Proinflammatory markers**			
TNF-α, ng/L	48.7 (-0.15, 104.8)	144.5 (113.2, 176.4)	***0.001*** ^**^
hs-CRP, μg/L	−53.1 (-318.3, 289.0)	578.6 (121.7, 1078.9)	***<0.001*** ^***^
sCD14, μg/L IL-6, ng/L	−6.7 (−25.9, 1.9) −1.6 (−3.5, 1.6)	−16.3 (−26.1, −5.7) 1.3 (−6.0, 4.7)	0.153 0.300

Bold italic ^*^p < 0.05; Bold italic ^**^p < 0.01; Bold italic^***^p < 0.001. The data were presented as median and IQR.

**Figure 2:**
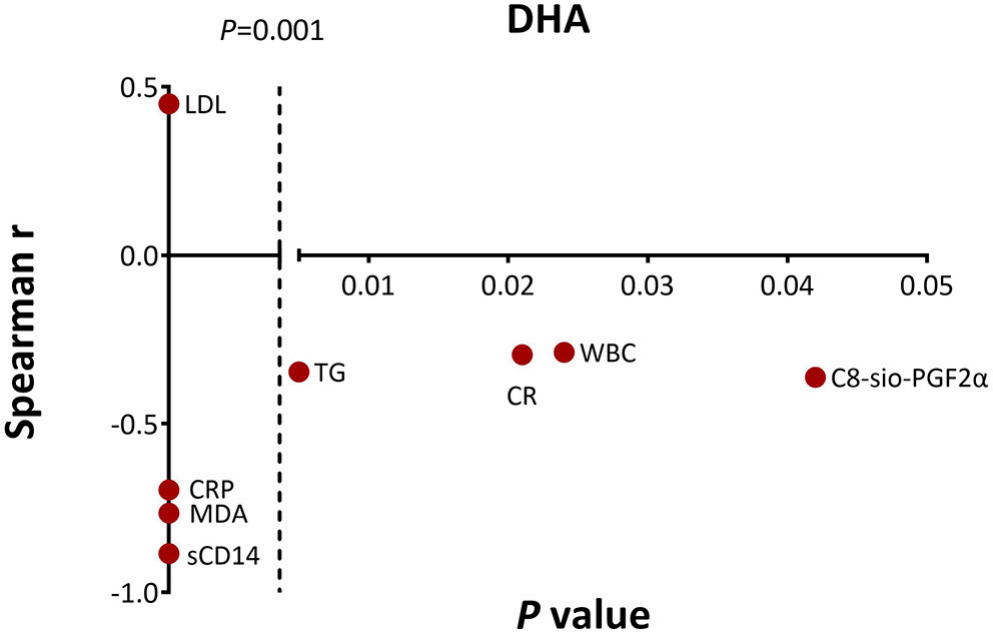
Significant associations between change in docosahexanoic acid (DHA) level and changes in clinical parameters, proinflammatory and oxidative stress markers as measured by the Spearman's correlations. CR, creatinine.

Similarly, in the DHA supplement group, the change in plasma DHA was negatively correlated with the level of TG (*r* = −0.346, *p* = 0.005), CRP (*r* = −0.696, *p* < 0.001), MDA (*r* = −0.765, *p* < 0.001), C8-sio-PGF2i (*r* = −0.362, *p* = 0.042), sCD14 (*r* = −0.885, *p* < 0.001), CR (*r* = −0.295, *p* = 0.021), and WBC (*r* = −0.288, *p* = 0.024). The increased level of DHA was positively correlated with increased level of LDL (*r* = 0.449, *p* < 0.001) (Figure 2).

### Effects of DHA Supplement on Gut Microbiota

**Figure 3:**
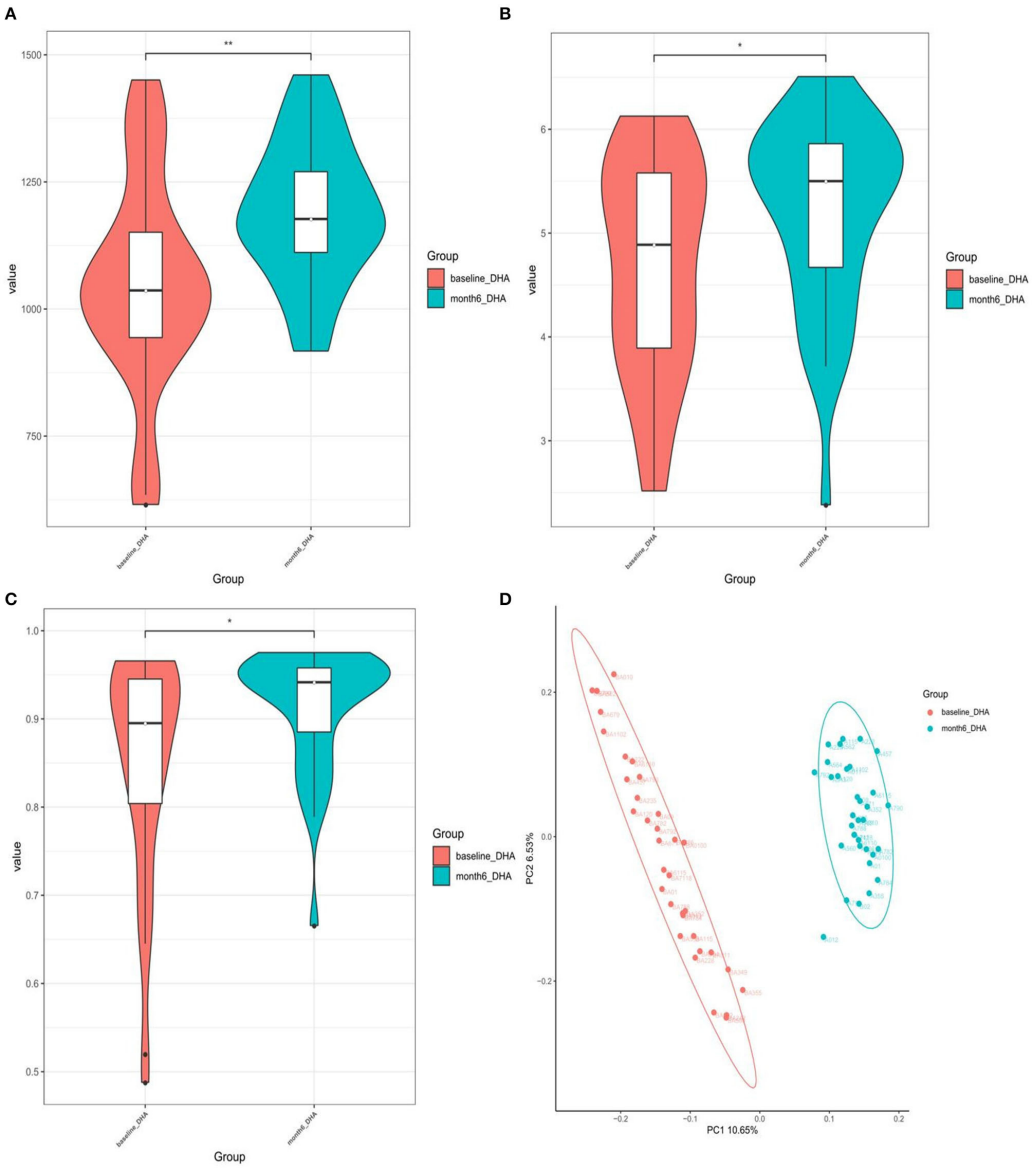
**(A)** Chao1-index; **(B)** Shannon-index; **(C)** Simpson-index; **(D)** unweighted analyses of similarities (ANOSIMs) and principal coordinates analysis (PCOA) based on the distance matrix of UniFrac dissimilarity of the fecal microbial communities. Each symbol represented a sample.

Based on the Shannon, Simpson, and Chao indices, the community diversity was significantly increased in the DHA supplement group compared with the baseline group [Chao index (*p* = 0.002), Shannon index (*p* = 0.037), and Simpson index (*p* = 0.015), Figures 3A–C]. The β-diversity of gut microbiota was evaluated based on the unweighted (*p* = 0.001) UniFrac distance matrix of the differences between the two groups in the fecal microbial communities (Figure 3D).

**Figure 4:**
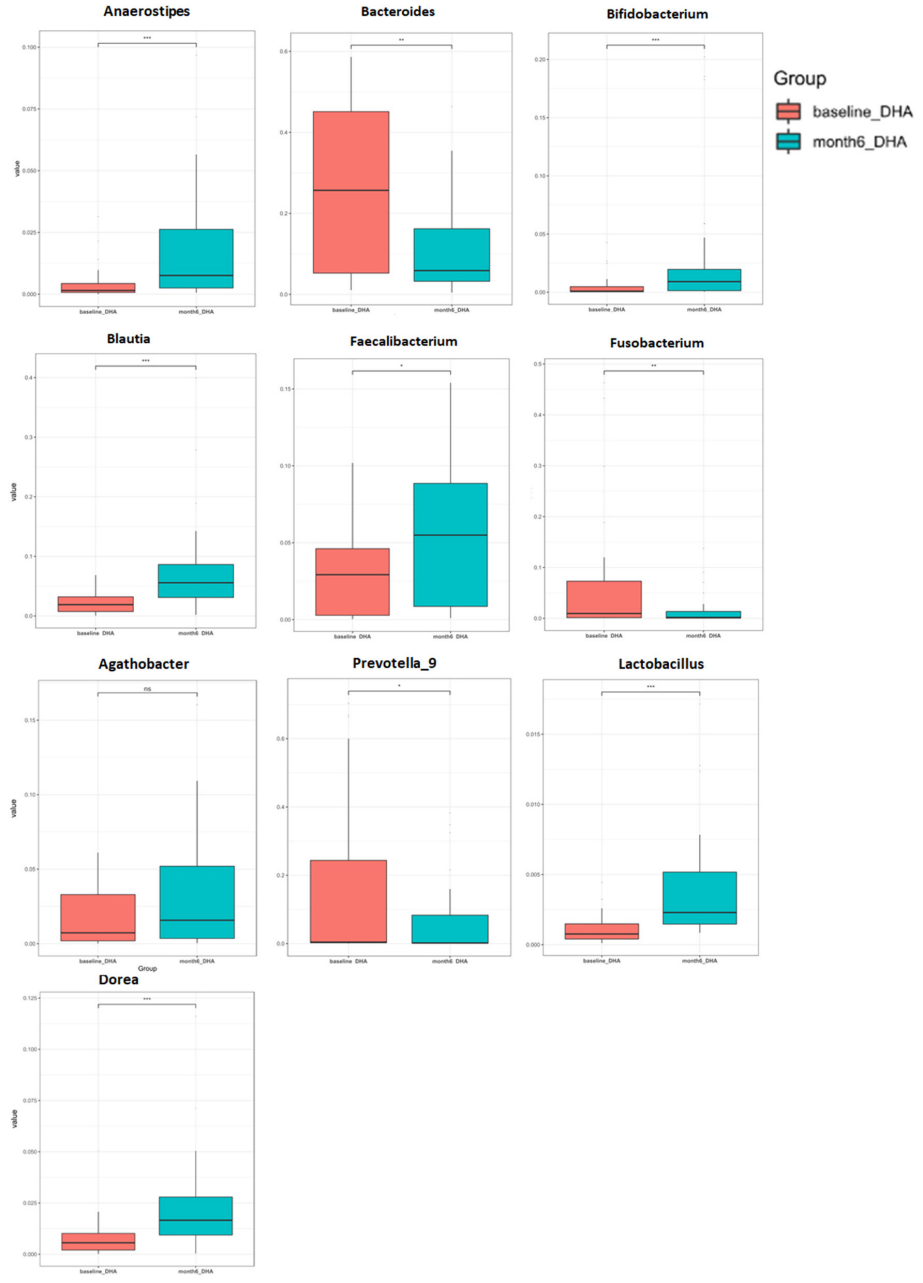
Changes of relative abundance of individual genera after DHA supplement.

One hundred sixty-two genera showed significant differences between the two groups. The top 10 most abundant genera are presented in Supplementary Table 4. We conducted the following correlation analyses using the top 10 most abundant difference genera. The DHA supplement led to a significant increase in the relative abundance of the bacterial genus including *Blautia, Bifidobacterium, Dorea, Lactobacillus, Faecalibacterium, Fusobacterium*, and *Agathobacter* after the intervention, but led to a reduction in *Bacteroides* and *Prevotella* (Figure 4 and Supplementary Table 4).

**Figure 5:**
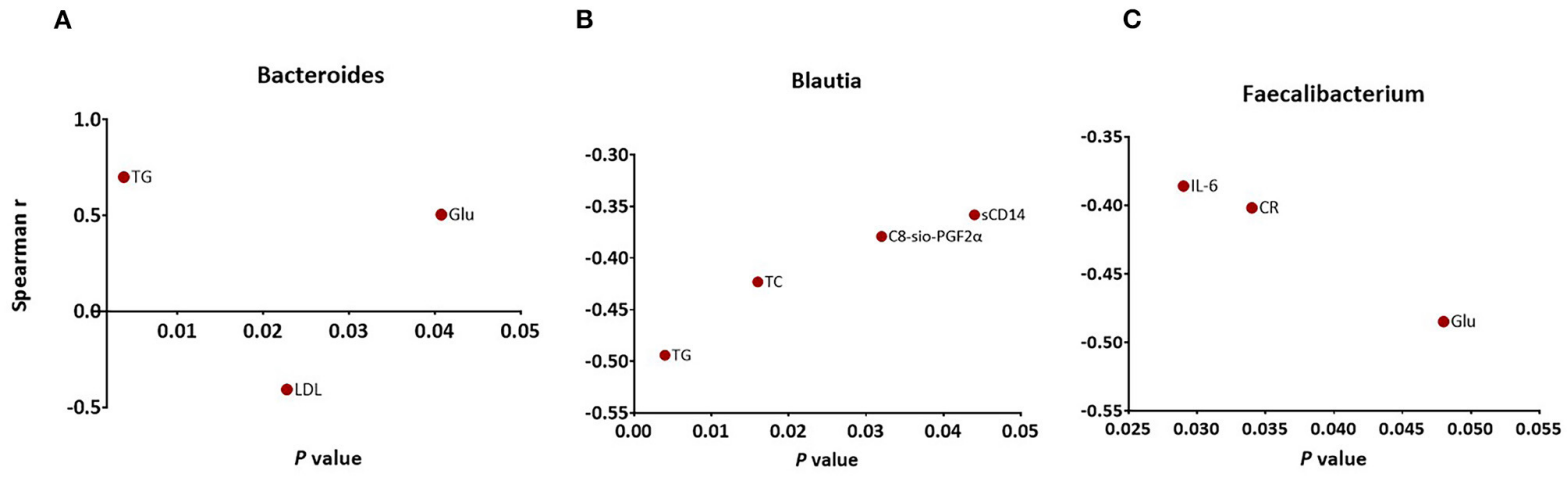
Significant associations between changes in specific markers and changes in abundance of **(A)** Bacteroides **(B)** Blautia **(C)** Faecalibacterium as measured by Spearman's correlations. FDR, false discovery rate.

The relative abundance of *Blautia* was negatively correlated with the level of TG (*r* = −0.494, *p* = 0.004), TC (*r* = −0.423, *p* = 0.016), sCD14 (*r* = −0.358, *p* = 0.044), and C8-sio-PGF2i (*r* = −0.379, *p* = 0.032), whereas the change in *Bacteroides* was positively correlated with the changes in TG (*r* = 0.701, *p* = 0.002), Glu (*r* = 0.505, *p* = 0.039) and negatively correlated with the changes in LDL (*r* = −0.407, *p* = 0.021). The change in relative abundance of *Faecalibacterium* was negatively associated with the changes in IL-6 (*r* = −0.386, *p* = 0.029), CR (*r* = −0.402, *p* = 0.034), and Glu (*r* = −0.485, *p* = 0.048) (Figure 5). Additionally, the relative abundance of *Agathobacter* (*r* = −0.488, *p* = 0.047) and Dorea (*r* = −0.517, *p* = 0.034) were negatively associated with the level of Glu (data not shown).

### Effects of DHA Supplement on Profiles of Fecal Metabolomic

**Figure 6:**
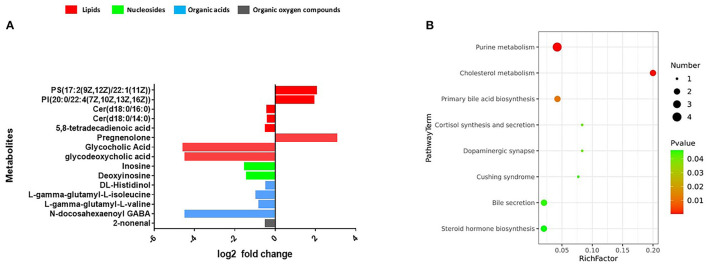
**(A)** Fecal metabolomics changed after the intervention. **(B)** The significantly perturbed pathways by the intervention.

We observed that the composition of fecal metabolites shifted significantly in response to the DHA supplement (*p* = 0.005) (Supplementary Figure 2). Multivariate and univariate statistical significance criteria (VIP > 1 and FDR < 0.05) were used to filter the metabolites that are different between the groups. In total, we identified 15 fecal metabolites that have been significantly changed. These metabolites include eight lipids, four amino acid metabolites, two nucleosides, and one organic oxygen compound after FDR correction (Supplementary Table 5). Among them, 12 were decreased [Cer(d18:0/14:0), Cer(d18:0/16:0), glycocholic acid, glycodeoxycholic acid, 5,8-tetradecadienoic acid, 2-nonenal, deoxyinosine, inosine, L-gamma-glutamyl-L-valine, L-gamma-glutamyl-L-isoleucine, N-docosahexaenoyl GABA, and DL-histidinol] and three were increased [PI (20:0/22:4(7Z,10Z,13Z,16Z), PS (17:2(9Z,12Z)/22:1(11Z), and pregnenolone] (Figure 6A).

**Figure 7:**
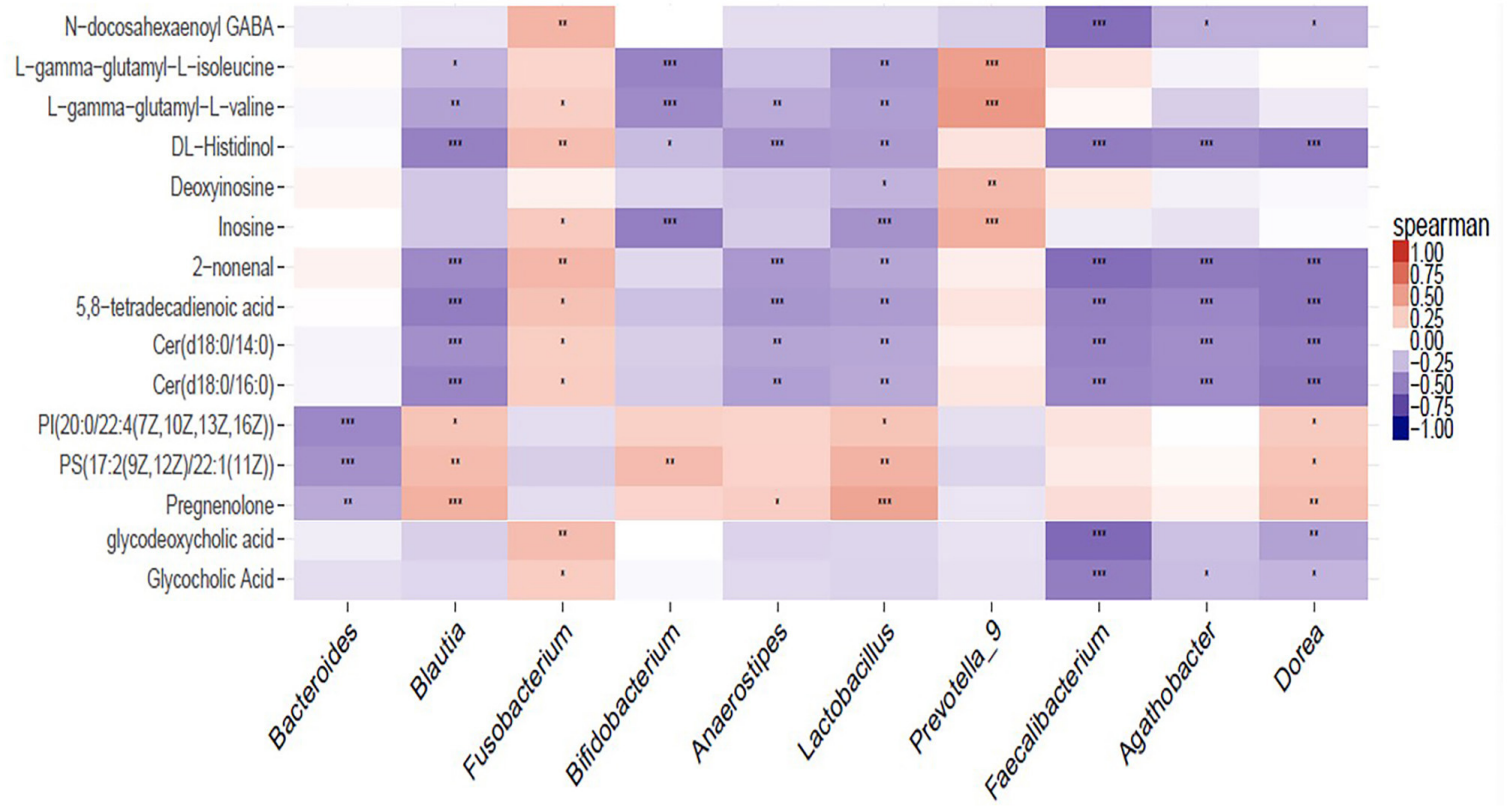
Heat map summarizing the correlation of perturbed gut microbiota genera and altered fecal metabolites. ^*^P < 0.05; ^**^P < 0.01; ^***^P < 0.001.

The significantly perturbed pathways are shown in Figure 6B. After FDR correction, there were significant differences in both the cholesterol metabolism (Rich factor = 0.200, *p* = 0.003) and purine metabolism (Rich factor = 0.042, *p* = 0.003) pathways. The changes in fecal concentrations of inosine and deoxyinosine were found to be positively correlated with the level of CD4 count (*r* = 0.35, *p* < 0.001) and WBC count (*r* = 0.28, *p* = 0.002), while no other significant associations were observed (data not shown). Multiple correlations were found between fecal metabolites and specific gut bacteria (Figure 7). The changes in metabolites that decreased in the DHA supplement group were consistently negatively correlated with the beneficial bacterial genera increased in the DHA group, such as *Blautia, Faecalibacterium*, and *Lactobacillus*.

## Discussion

Our study is the first randomized, double blind, placebo-controlled trial that demonstrated a beneficial effect of DHA supplement on gut microbiota, a profile of fecal metabolomics, proinflammatory and oxidative stress factors in HIV-infected patients with NCI. Our findings suggest that the DHA supplement has overall favorable effects on several biomarkers, and these DHA benefits may be due to their ability to modulate gut microbiota.

Some studies found a significant improvement in cognition and memory in healthy and mild cognitive impairment patients after supplementation with DHA (18). However, we did not observe any significant increase in GDS or MMSE scores in the HIV-infected patients in response to DHA supplementation. This may be attributed to the strict subject exclusion criteria which excluded patients with cardiovascular and cerebrovascular diseases. Previous studies have suggested that the positive action of DHA on the brain is mediated by their beneficial effects on a range of anti-inflammatory processes facilitating vasodilation (25). For example, their regulation of the vascular tone *via* the increase in NO production may lead to a rise in cerebral blood perfusion. We found the beneficial effect of DHA on blood lipid markers. The positive effects of n-3 PUFA supplementations (mainly DHA as fish oil) on decreasing TG are well-established in HIV-infected patients. For instance, randomized clinical trials which treat hypertriglyceridemia in HIV-infected patients with n-3 PUFA demonstrated its beneficial effect in decreasing TG levels from 10 to 56% of the baseline values (26–28). Previous studies found that HIV-infected patients had increased levels of LDL (from 5 to 20%) in response to the treatment with fish oil (28, 29). In agreement with these clinical trials, the HIV-infected patients with NCI had a significantly decreased level of fasting TG after receiving supplementation with DHA 3.15 g daily. Furthermore, TG decrease and LDL cholesterol increase were related to the increasing DHA levels.

In addition, we found that WBC and plasma concentrations of TNF-α, hs-CRP were significantly decreased after DHA supplement, WBC and sCD14 decreases were related to the increasing DHA levels. However, in HIV-infected patients, whether PUFA has an effect on inflammation is controversial. Some studies showed that PUFA can decrease hs-CRP, IL-6, and TNF-α circulating levels and thus has a beneficial effect, whereas others show no benefit at all (30, 31). There is a well-known interplay between oxidative stress and inflammation (32). However, there is a lack of favorable reports on the effects of DHA supplementation on oxidative stress in patients with HIV infection. A recent clinical trial found no differences between the n-3 PUFA and the placebo group on oxidative stress in patients with HIV infection (33). We found the change in plasma DHA was negatively correlated with the serum levels of MDA and C8-sio-PGF2i. Taken together, although we did not observe the benefit of DHA on cognitive performance, our results suggest a possible beneficial effect of DHA by reducing TG, inflammatory cytokines, and markers for oxidative stress.

HIV-infected patients with NCI showed a low microbiota diversity in our previous study (manuscript submitted). Higher gut microbiome diversity is linked to lower inflammation (34). In this study, DHA supplement improved the microbial α-diversity which reinforced the notion that DHA is linked to lower gut inflammation. In addition, accumulating evidence found that DHA showed a positive regulatory role in the gut microbiota community (7, 35, 36). For example, a clinical trial found a reversible increase in the abundance of “beneficial” bacterial genera, including *Bifidobacterium, Lachnospira, Roseburia*, and *Lactobacillus* in 25 volunteers who were given 3.5 g DHA for 30 days (36). In line with previous work, we found that *Bifidobacterium, Blautia, Faecalibacterium, Lactobacillus*, genera which were known to produce short-chain fatty acid (SCFA), were increased in the DHA group. This is also consistent with data obtained in mice (37, 38). We identified several bacterial genera that have potential implications in the host metabolic health with correlation analysis. We observed similar negative associations between *Blautia* and TC and non-HDL-C which is consistent with previous studies (39, 40), we observed a similar negative correlation between *Blautia* and blood lipid profiles. Furthermore, we found a negative correlation between the abundance of *Blautia* and *Faecalibacterium* and proinflammatory and oxidative stress markers. Therefore, the increased abundances of *Blautia* and *Faecalibacterium* in the DHA group suggest that the change in gut microbiota may impact the host metabolic health in patients with NCI.

Although our study did not observe cognitive improvement with DHA on the intervention of NCI with NP test, markers of neural injury could be used to better assess the patients in a more sensitive manner (41). For example, ceramides have been found to accumulate in HIV-infected patients with progressive NCI. Therefore, ceramides may be used as indicators for neurological dysfunction (42). Bile acids (BAs) were also found to have a role in influencing cognitive function. For example, increasing evidence suggests BAs could serve as biomarkers of Alzheimer's disease (AD) and cognitive aging (43, 44). Microbially derived toxic BAs are heightened in patients with Parkinson's disease (PD) or AD (45, 46). In line with these results, DHA supplement significantly decreased the fecal concentrations of ceramides [Cer(d18:0/16:0 and Cer(d18:0/14:0)] and BAs (glycocholic acid and glycodeoxycholic acid). In addition, to reduce the BAs metabolites, DHA induced significant alterations in the pathway of cholesterol metabolism. Recent epidemiological and molecular studies have linked the disorders of cholesterol metabolism to increased risks for developing neurodegenerative diseases, such as PD, AD, Huntington's disease (HD), and other cognitive deficits that arise at old age (47). DHA also induced significant alteration in a pathway of purine metabolism. We found that the fecal concentrations of inosine and deoxyinosine were decreased in the DHA group which is consistent with the pathway enrichment analysis. Homeostatic imbalance of purine metabolism has been reported to be related to neurotoxicity in schizophrenia (48, 49). Thus, our data suggest that DHA may have a neuroprotective effect by altering fecal metabolomics profiles. In contrast with previous studies which identified markers of excitotoxicity associated with HIV-associated neurocognitive disorders (HAND) pathogenesis, including the amino acid precursors of serotonin and dopamine and neurotransmitter glutamate (41), we observed significantly decreased fecal concentrations of inhibitory neurotransmitter GABA and several other amino acid metabolites in the DHA group, which need to be confirmed in the future study.

In addition, ceramides, BAs, 5,8-tetradecadienoic acid, 2-nonenal are considered as lipid markers of oxidative stress (42). Inosine is assumed to accumulate as a result of excessive oxidative processes. Purine catabolism mediates the mitochondrial response to oxidant stress to maintain homeostasis (49). Increased markers of oxidative stress are linked to the HAND and cognitive impairment (41). Thus, the reduction of these metabolites in the DHA group suggests that DHA supplements might reduce oxidative stress *via* the gut microbiota. In addition, previous studies also found that ceramides accumulated in HIV positive patients with progressive neurocognitive impairment (41). These consistent findings suggest that ceramides may be sensitive indicators of neurological dysfunction. Furthermore, the positive associations between changes in inosine and deoxyinosine concentrations and the changes in plasma levels of inflammatory cells, such as CD4 T cells and WBC, indicated a potential mechanism through which the DHA supplement also reduces inflammation *via* the gut microbiota. Consistently, these mentioned inflammation and oxidative stress related metabolites were negatively associated with the beneficial bacterial genera, including *Blautia, Faecalibacterium*, and *Lactobacillus*. Thus, our results reinforce that the protective effect of DHA was attributed to its regulatory effect on beneficial bacteria.

This study had a limited chance for the collection of blood and fecal samples which was only at baseline and at the end of the trial. Another limitation was the relatively small sample size of this trial which warrants caution in the interpretation of this dataset. Third, although patients received instruction on maintaining their diet habits, and there is no difference within two intervention arms, we could not fully exclude the confounding effect of diet might have on gut microbiota.

## Conclusion

In summary, we did not observe any significant increase in GDS or MMSE scores in the HIV-infected patients in response to DHA supplementation. DHA supplementation influences the gut microbiota and profiles of fecal metabolomics which contribute to reducing proinflammatory and oxidative stress factors for HIV infected patients with NCI.

## Data Availability Statement

The original contributions presented in the study are included in the article/Supplementary Material, further inquiries can be directed to the corresponding author/s.

## Ethics Statement

The studies involving human participants were reviewed and approved by Institutional Review Board (IRB) of the School of Public Health at Fudan University (no.IRB#2019-06-0759). The patients/participants provided their written informed consent to participate in this study.

## Author Contributions

RD and NH proposed and developed the research question, wrote, reviewed, and edited the manuscript. HL and NH generally supervised the study. RD, YD, XC, RS, SY, JL, BZ, XX, and KW contributed to data collection and data management. WS contributed to laboratory management and tests. RD performed data analysis. YD critically reviewed the manuscript. DD provided expertise on neuropsychological tests. All authors read and approved the final manuscript.

## Funding

This study was supported by the National Natural Science Foundation of China (81773485), the China National Science and Technology Major Projects on Infectious Diseases (2018ZX10721102-004), and partially supported by the Shanghai Municipal Health Commission (GWV-10.1-XK16).

## Conflict of Interest

The authors declare that the research was conducted in the absence of any commercial or financial relationships that could be construed as a potential conflict of interest.

## Publisher's Note

All claims expressed in this article are solely those of the authors and do not necessarily represent those of their affiliated organizations, or those of the publisher, the editors and the reviewers. Any product that may be evaluated in this article, or claim that may be made by its manufacturer, is not guaranteed or endorsed by the publisher.
