# Supplementary material for: Effects of Docosahexanoic Acid on Gut Microbiota and Fecal Metabolites in HIV-Infected Patients With Neurocognitive Impairment: A 6-Month Randomized, Double-Blind, Placebo-Controlled Trial
Source: Front Nutr. 2022 Jan 21;8:756720. doi: 10.3389/fnut.2021.756720 (PMC8814435; doi:10.3389/fnut.2021.756720)
Supplement: Supplementary file 1 [file Data_Sheet_1.docx]

Supplementary Material

# Supplemental Experimental Procedures

**16S rRNA sequencing**

***DNA extraction***

Total genomic DNA was extracted using DNA Extraction Kit following the manufacturer’s instructions. Quality and quantity of DNA was verified with NanoDrop and agarose gel. Extracted DNA was diluted to a concentration of 1 ng/μl and stored at -20 °C until further processing. The diluted DNA was used as template for PCR amplification of bacterial 16S rRNA genes with the barcoded primers and Takara Ex Taq (Takara). For bacterial diversity analysis, V3-V4 (or V4-V5) variable regions of 16S rRNA genes was amplified with universal primers 343 F and 798 R (or 515F and 907R for V4-V5 regions).

***Library Construction***

Amplicon quality was visualized using gel electrophoresis, purified with AMPure XP beads (Agencourt), and amplified for another round of PCR. After purified with the AMPure XP beads again, the final amplicon was quantified using Qubit dsDNA assay kit. Equal amounts of purified amplicon were pooled for subsequent sequencing.

***Bioinformatic analysis***

Raw sequencing data were in FASTQ format. Paired-end reads were then preprocessed using Trimmomatic software to detect and cut off ambiguous bases (N). It also cut off low quality sequences with average quality score below 20 using sliding window trimming approach. After trimming, paired-end reads were assembled using FLASH software. Parameters of assembly were: 10bp of minimal overlapping, 200bp of maximum overlapping and 20% of maximum mismatch rate. Sequences were performed further denoising as follows: reads with ambiguous, homologous sequences or below 200bp were abandoned. Reads with 75% of bases above Q20 were retained. Then, reads with chimera were detected and removed. These two steps were achieved using QIIME software (version 1·8·0). Clean reads were subjected to primer sequences removal and clustering to generate operational taxonomic units (OTUs) using Vsearch software with 97% similarity cutoff. The representative read of each OTU was selected using QIIME package. All representative reads were annotated and blasted against Silva database Version 123 (or Greengens) (16s/18s rDNA) using RDP classifier (confidence threshold was 70%). All representative reads were annotated and blasted against Unite database (ITSs rDNA) using blast.

**Measurement of faecal Metabolomics**

***Chemicals***

All chemicals and solvents were analytical for HPLC grade. Water, methanol, acetonitrile, formic acid were purchased from CNW Technologies GmbH (Düsseldorf, Germany). L-2 chlorophenylalanine was from Shanghai Hengchuang Bio-technology Co., Ltd. (Shanghai, China).

***Sample Preparation***

The sample was transferred to a 1·5-mL Eppendorf tube. Two small steel balls were added to the tube. 20 μL internal standard (2-chloro-l-phenylalanine in methanol, 0·3 mg/mL) and extraction solvent with methanol /water (4/1, v/v) were added to each sample. Samples were stored at -20 °C for 5 min and then grinded at 60 HZ for 2 min, ultrasonicated at ambient temperature (25 °C to 28 °C) for 10 min, stored at -20 °C for 30 min. The extract was centrifuged at 13000 rpm, 4 °C for 15 min. The supernatant in a glass vial was dried in a freeze concentration centrifugal dryer. The mixture of methanol and water (1/4, vol/vol) were added to each sample, samples vortexed for 30 s, then placed at 4°C for 2 min. Samples were centrifuged at 13000 rpm, 4 °C for 5 min. The supernatants from each tube were collected using crystal syringes, filtered through 0·22 μm microfilters and transferred to LC vials. The vials were stored at 4°C until LC -MS analysis. QC samples were prepared by mixing aliquots of the all samples to be a pooled sample.

***Data Preprocessing and Statistical Analysis***

The acquired LC-MS raw data were analyzed by the progenesis QI software (Waters Corporation，Milford, USA) using the following parameters. Precursor tolerance was set 5 ppm, fragment tolerance was set 10 ppm, and retention time (RT) tolerance was set 0·02 min. Internal standard detection parameters were deselected for peak RT alignment, isotopic peaks were excluded for analysis, and noise elimination level was set at 10·00, minimum intensity was set to 15 % of base peak intensity. The Excel file was obtained with three-dimension data sets including m/z, peak RT and peak intensities, and RT–m/z pairs were used as the identifier for each ion. The resulting matrix was further reduced by removing any peaks with missing value (ion intensity = 0) in more than 50 % samples. The internal standard was used for data QC (reproducibility).

Metabolites were identified by progenesis QI (Waters Corporation, Milford, USA) Data Processing Software, based on public databases such as http://www.hmdb.ca/; http://www.lipidmaps.org/ and self-built databases. The positive and negative data were combined to get a combine data which was imported into R ropls package. Principle component analysis (PCA) and (orthogonal) partial least-squares-discriminant analysis (O)PLS-DA were carried out to visualize the metabolic alterations among experimental groups, after mean centering (Ctr) and Pareto variance (Par) scaling, respectively. The Hotelling’s T2 region, shown as an ellipse in score plots of the models, defines the 95% confidence interval of the modeled variation. Variable importance in the projection (VIP) ranks the overall contribution of each variable to the OPLS-DA model, and those variables with VIP > 1 are considered relevant for group discrimination.

In this study, the default 7-round cross-validation was applied with 1/seventh of the samples being excluded from the mathematical model in each round, in order to guard against overfitting. The differential metabolites were selected on the basis of the combination of a statistically significant threshold of variable influence on projection (VIP) values obtained from the OPLS- DA model and *p* values from a two-tailed Student’s t test on the normalized peak areas, where metabolites with VIP values larger than 1·0 and *p* values less than 0·05 were considered as differential metabolites.

# Supplementary Figures and Tables


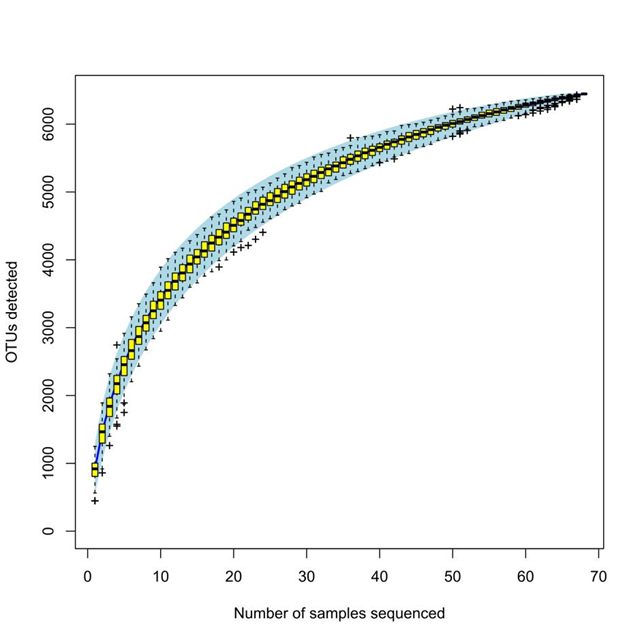


Figure S1. The accumulation curve related to 16S rRNA gene sequencing

**Table S1.** The fatty acid composition and the content (g/100g) of DHA algal oil.

| C14:0 | C15:0 | C16:0 | C16:1 | C17:0 | C17:1 | C18:0 | C18:1 | C18:2 | C18:3 | C20:3 | C22:0 | C22:6 |
| --- | --- | --- | --- | --- | --- | --- | --- | --- | --- | --- | --- | --- |
| 0.591 | 0.885 | 28.161 | 0.271 | 1.300 | 0.251 | 1.759 | 0.597 | 1.429 | 0.165 | 0.662 | 0.495 | 45.000 |

**Table S2. The manufacture and code number of each commercial kit used.**

| Commercial kit | Manufacture | Code number |
| --- | --- | --- |
| Scd14 ELISA | Shanghai Enzyme-linked Biotechnology | YY26133 |
| CRP ELISA | Shanghai Enzyme-linked Biotechnology | YY36854 |
| IL-6 ELISA | Shanghai Enzyme-linked Biotechnology | YY26634 |
| TNF-α ELISA | Shanghai Enzyme-linked Biotechnology | YY033532 |
| MDA ELISA | Shanghai Enzyme-linked Biotechnology | YY698211 |
| 8-isoprostane F2α ELISA | Shanghai Enzyme-linked Biotechnology | YY236846 |
| LDHA ELISA | Shanghai Enzyme-linked Biotechnology | YY569414 |

**Table S3. Dietary nutrient intake after the intervention.**

| **Characteristics** | **DHA group (n = 35)** | **Placebo group (n = 33)** | ***P* values** |
| --- | --- | --- | --- |
| Energy, kcal/d  Carbohydrate, g/d  Protein, g/d  Fat, g/d  Dietary fiber, g/d  Cholesterol, g/d | 1059.6 (729.3, 1439.3)  163.4 (131.5, 203.6)  47.3 (31.1, 67.3)  29.2 (13.6, 53.5)  8.7 (5.6, 11.9)  294.4 (119.6, 428.6) | 1296.4 (867.9, 1561.2)  182.6 (137.4, 222.5)  53.8 (36.9, 72.8)  33.4 (22.1, 58.6)  11.0 (6.3, 13.9)  345.1 (175.2, 475.9) | 0.328  0.412  0.197  0.372  0.312  0.344 |

**Table S4. Altered gut microbiota compositions.**

| Altered microbiota | *P* value | ^a^ Adjusted *P* value |
| --- | --- | --- |
| Blautia ↑ | ***<0.001****** | ***0.008***** |
| Bifidobacterium ↑ | ***<0.001****** | ***0.008***** |
| Dorea ↑ | ***<0.001****** | ***0.008***** |
| Anaerostipes ↑ | ***<0.001****** | ***0.008***** |
| Lactobacillus↑ | ***<0.001****** | ***0.008***** |
| Bacteroides ↓ | ***0.004***** | ***0.025**** |
| Faecalibacterium ↑ | ***0.011**** | 0.050 |
| Fusobacterium ↑ | ***0.011**** | 0.050 |
| Prevotella_9 ↓ | ***0.024**** | 0.090 |
| Agathobacter ↑ | ***0.019**** | 0.352 |

↑: up-regulated; ↓: down-regulated; Bold italic ^*^*P* < 0.05; Bold italic ^**^*P* < 0.01;

^a^ Adjusted *P* value: *P*-values were adjusted to control the false discovery rate (FDR).


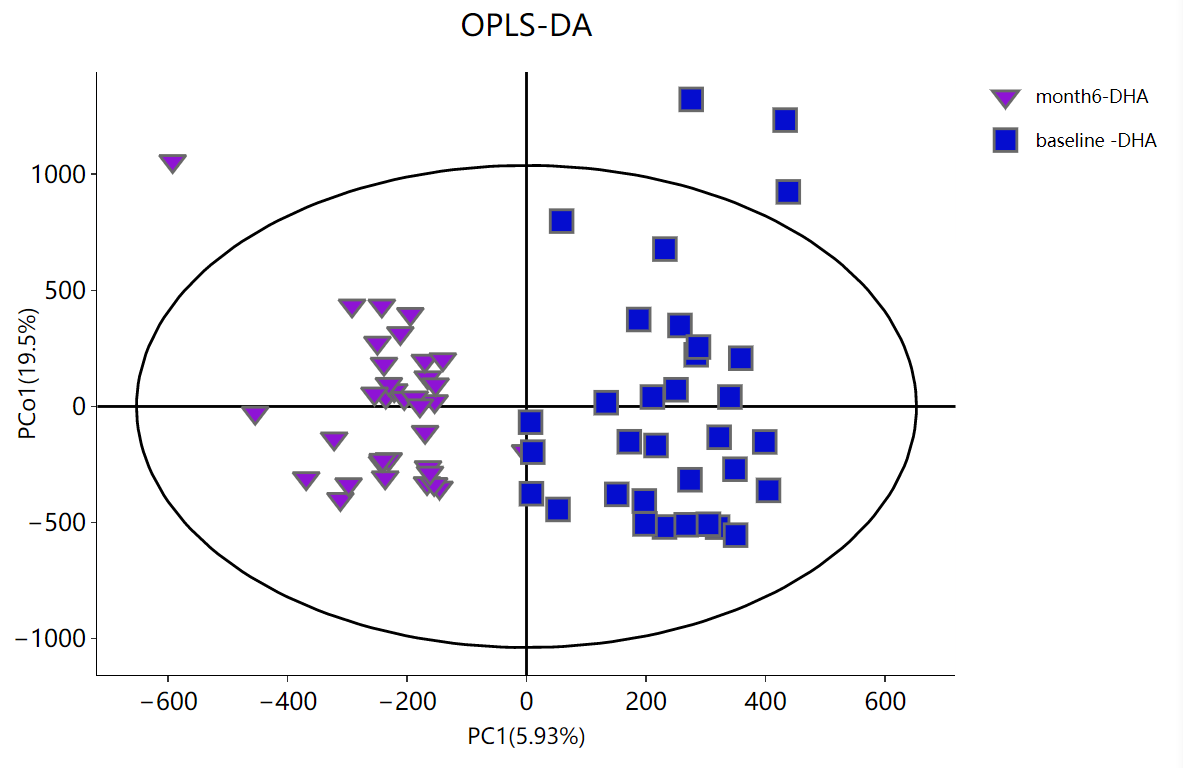


Figure S2. OPLS-DA score plots of faecal samples from the baseline and DHA supplement groups.

Table S5. Differentiating metabolites identified from the data set

| **Metabolites** | **Class** | **Sub Class** | ***P-value*** | **^b^ Adjusted *P value*** | **^a^ FC** |
| --- | --- | --- | --- | --- | --- |
| PS (17:2(9Z,12Z)/22:1(11Z)) | Glycerophospholipids | Glycerophosphoserines | 9.3×10^-9^ | 2×10^-6^ | 4.21312 |
| PI (20:0/22:4(7Z,10Z,13Z,16Z)) | Glycerophospholipids | Glycerophosphoinositols | 2.4×10^-7^ | 2.3×10^-5^ | 3.87015 |
| Cer (d18:0/16:0) | Sphingolipids | Ceramides | 0.00608 | 0.04611 | 0.73531 |
| Cer (d18:0/14:0) | Sphingolipids | Ceramides | 0.0083 | 0.07 | 0.74659 |
| Glycocholic Acid | Steroids and steroid derivatives | Bile acids, alcohols and derivatives | 0.00431 | 0.04342 | 0.04128 |
| glycodeoxycholic acid | Steroids and steroid derivatives | Bile acids, alcohols and derivatives | 0.00468 | 0.04596 | 0.04458 |
| Pregnenolone | Steroids and steroid derivatives | Pregnane steroids | 2.4×10^-7^ | 2.3×10^-5^ | 8.50998 |
| 5,8-tetradecadienoic acid | Fatty Acyls | Fatty acids and conjugates | 0.001 | 0.01552 | 0.70487 |
| 2-nonenal | Organooxygen compounds | Carbonyl compounds | 0.00088 | 0.01439 | 0.70348 |
| Deoxyinosine | Purine nucleosides | Purine 2'-deoxyribonucleosides | 0.00105 | 0.01597 | 0.36828 |
| Inosine | Purine nucleosides | Unclassified | 0.00194 | 0.02497 | 0.34619 |
| L-gamma-glutamyl-L-valine | Carboxylic acids and derivatives | Amino acids, peptides, and analogues | 0.00234 | 0.02849 | 0.56151 |
| L-gamma-glutamyl-L-isoleucine | Carboxylic acids and derivatives | Amino acids, peptides, and analogues | 0.00367 | 0.03896 | 0.50671 |
| N-docosahexaenoyl GABA | Carboxylic acids and derivatives | Amino acids, peptides, and analogues | 0.00473 | 0.04262 | 0.04442 |
| DL-Histidinol | Carboxylic acids and derivatives | Amino acids, peptides, and analogues | 0.00187 | 0.02429 | 0.71687 |

^a^ FC: Fold change, with a value larger than 1 indicates a higher level of the metabolite after DHA supplement; Fold change value lower than 1 indicates a lower level of the metabolite after DHA supplement. ^b^ Adjusted *P* value: *P*-values were adjusted to control the false discovery rate (FDR).
